# Determining the value of the abdominal core health quality collaborative to support regulatory decisions

**DOI:** 10.1007/s10029-024-02990-5

**Published:** 2024-04-29

**Authors:** B. K. Poulose, E. Avila-Tang, H. Schwartzman, T. Bisgaard, L. N. Jørgensen, G. Gibeily, A. Schick, D. Marinac-Dabic, M. J. Rosen, G. Pappas

**Affiliations:** 1 Center for Abdominal Core Health, The Ohio State University Wexner Medical Center, Columbus, OH USA; 2 United States Food and Drug Administration, Silver Spring, MD USA; 3 Abdominal Core Health Quality Collaborative Foundation, Centennial, CO USA; 4 Surgical Department, Zealand University Hospital, Køge, Roskilde, Denmark; 5 Digestive Disease Center, Bispebjerg Hospital, University of Copenhagen, Copenhagen, Denmark; 6 Center for Abdominal Core Health, The Cleveland Clinic, Cleveland, OH USA

**Keywords:** Hernia, ACHQC, Regulatory, Post-market surveillance

## Abstract

**Purpose:**

The study objective is to document value created by real-world evidence from the Abdominal Core Health Quality Collaborative (ACHQC) for regulatory decisions. The ACHQC is a national effort that generates data on hernia repair techniques and devices.

**Methods:**

Two retrospective cohort evaluations compared cost and time of ACHQC analyses to traditional postmarket studies. The first analysis was based on 25 reports submitted to the European Medicines Agency of 20 mesh products for post-market surveillance. A second analysis supported label expansion submitted to the Food and Drug Administration, Center for Devices and Radiological Health for a robotic-assisted surgery device to include ventral hernia repair. Estimated costs of counterfactual studies, defined as studies that might have been done *if the registry had not been available*, were derived from a model described in the literature. Return on investment, percentage of cost savings, and time savings were calculated.

**Results:**

45,010 patients contributed to the two analyses. The cost and time differences between individual 25 ACHQC analyses (41,112 patients) and traditional studies ranged from $1.3 to $2.2 million and from 3 to 4.8 years, both favoring use of the ACHQC. In the second label expansion analysis (3,898 patients), the estimated return on investment ranged from 11 to 461% with time savings of 5.1 years favoring use of the ACHQC.

**Conclusions:**

Compared to traditional postmarket studies, use of ACHQC data can result in cost and time savings when used for appropriate regulatory decisions in light of key assumptions.

## Introduction

Use of mesh in ventral and inguinal hernia repair has been shown to significantly decrease the incidence of recurrent hernia [1, 2]. This benefit is offset by mesh-related complications including chronic pain, infection, bowel obstruction, and fistulization [3]. Concern about safety and effectiveness of mesh used in hernia repair has been documented in published literature, regulatory action, and litigation [4–6]. Weaknesses in effective post-market surveillance of hernia mesh and related products are also well understood [7, 8]. The European Medicines Agency (EMA) recently increased requirements for post-market surveillance data for medical devices including hernia mesh [9]. Clinical registries have been used to augment traditional sources of data to guide clinical practice, research, and regulatory decision making [10]. Evaluation of data from the Danish Ventral Hernia Database and German HerniaMed database led to the voluntary withdrawal of a mesh device from the global market due to unacceptable rates of recurrence when used in laparoscopic ventral hernia repair [11].

Access to evidence about safety, effectiveness, and quality of medical devices is essential to inform care—a goal shared by stakeholders including patients, clinicians, health systems, payers, device manufacturers, and regulators. In the United States, the Food and Drug Administration does require post-market surveillance of hernia mesh and devices used in hernia care (e.g. fixation devices, robotics). This surveillance currently consists of the Manufacturer and User Facility Device Experience (MAUDE) database and the MedWatch system which allows health professionals, patients, and consumers to report adverse events. Supplementing this information through traditional means of evidence generation for device evaluation is very costly, time consuming, and has limitations [12–14]. Real-world evidence (RWE) based on routinely collected clinical data is increasingly being harnessed to support robust, affordable, and timely evidence generation [15]. The FDA has initiated several programs to improve post-market surveillance of medical devices based on RWE. The Medical Product Safety Network (MedSun) was established in 2002 with the primary goal to work collaboratively with the clinical community to identify, understand, and solve problems with the use of medical devices. This enhanced national medical device surveillance network includes 350 hospitals nationwide and engages in bi-directional interactions between FDA and hospitals, targeted surveys and problem solving to help detect and/or amplify potential signals. MedSun participants also voluntarily report problems with devices, such as ‘close-calls,’ potential for harm, and other safety concerns, thus enabling proactive actions to correct the problem before the adverse event occurs. The Medical Device Innovation Consortium (MDIC) and its program, the National Evaluation System for health Technology Coordinating Center (NESTcc) in partnership with FDA have been developing an active surveillance system of electronic health data to better understand and act upon the safety of medical devices used in a real-world setting. The active surveillance program is focused on advancing data capture and analysis to enable FDA to act upon the findings in a timely manner. The Coordinated Registry Networks (CRNs), as proposed by the National Medical Device Registry Task Force (NMDRTF), strategically bring together RWE from a variety of sources to support improved device evaluation [16].

The hypothesis of this study is that Abdominal Core Health Quality Collaborative (ACHQC) data used for regulatory purposes result in cost and time savings compared to traditional studies performed for regulatory approval.

The objective of this study is to determine the value created by the ACHQC for regulatory decision-making concerning medical devices used in abdominal wall hernia repair. We compared use of the ACHQC registry to studies that *might have been conducted* had the registry not existed (counterfactual study).

## Methods

### Data sources and approach

The ACHQC is a nonprofit, national quality improvement organization with mission to maximize the quality and value of health care for patients who suffer from hernia disease and diseases of the abdominal wall or abdominal core [17]. The ACHQC collects clinical information, long-term follow-up data and patient reported outcomes related to hernia repair and abdominal core surgery. The ACHQC is a Centers for Medicare & Medicaid Services Qualified Clinical Data Registry and is an authoritative resource for organizations to assess quality metrics and demonstrate a commitment to efficient, value-based patient-centered care for the hernia patient. Ethical approval and performance of this study was approved by The Ohio State Wexner Medical Center Institutional Review Board (IRB); informed consent was not required as this study was granted exempt status as the secondary analysis of aggregate, deidentified pre-existing data.

The ACHQC registry collects data on procedures performed in 50 states and approximately equally from academic and non-academic settings with 465 participating surgeons and a total enrollment of 100,043 patients [18]. The registry participating sites collect 30-day postoperative follow-up data; 95% of current participating centers provide this short-term follow-up. Routine long-term (1 year or greater) longitudinal data are collected by some participants and via patient reported outcomes; this results in available long-term follow-up of 15–20% across the collaborative. ACHQC currently collects data on 2% of abdominal hernia operations annually in the United States. There is a capability to add additional targeted long-term follow-up performed through a combination of electronic medical record review and direct contact of patients, increasing long-term follow-up [18, 19]. The costs incurred by medical device manufacturers as ACHQC Foundation Partners include overhead costs and costs associated with individual device (mesh) reports.

This manuscript presents two analyses comparing cost and time required to perform these evaluations in the registry compared to traditional studies. Metrics for these analyses include calculation of return on investment, percent cost saved, and time saved.

The first analysis is based on 25 reports to the EMA of 20 mesh products from three hernia mesh manufacturers using ACHQC data to meet new EMA post-market surveillance requirements. These analyses included de-identified patient demographics, comorbid conditions, 30-day and long-term outcomes including complications, wound events, recurrence, and quality of life. The cost and time needed to conduct these analyses were compared to the cost and time needed to conduct traditional studies that would have been needed if the registry had not been used. Since the new EMA post-market surveillance data requirements do not approximate traditional post-approval studies (PAS) and the reports to the EMA varied widely in the number of cases studied and registry follow-up time, and these ACHQC analyses do not approximate traditional PAS, they can only be compared to counterfactual cases offered in a general way.

The second analysis draws from data of the ACHQC used to support a label expansion request for a robotic-assisted surgery device. In this case, the company contracted with the ACHQC to evaluate off-label use of their device for the company to justify marketing claims indicating device use for ventral hernia repair. The ACHQC analyses included de-identified data on patient demographics, comorbid conditions, and 30-day outcomes including complications, wound events, and recurrence. Based on a review of registry data provided from 2013 to 2017, the registry data met or exceeded the clinical data recommended by FDA with respect to number of patients, number of sites, patient demographics and comorbidities, duration of follow-up, and number of follow-up visits typically provided to justify 510(k) hernia mesh device clearances. These data are publically available in the 510(k) summary information published on the FDA webpage [20]. This comparison allows formal return on investment (ROI) and time savings (TS) calculation to be performed.

### Costs of counterfactual studies

The counterfactual study (i.e., the study that might have been conducted had the registry not existed) served as the main comparator for the actual analyses performed using ACHQC data. We calculated an estimated cost of each counterfactual study using a model described by Wimmer et al.[21] Cost drivers in the Wimmer et al. model include study size; number of sites; need for recruitment; randomization or control groups; percentage of sites outside the US; length of follow-up (years); number of patient evaluations per year; patient evaluation type (phone/in-person); follow-up procedure required (yes/no), if yes, total procedure count and type of procedure (imaging/invasive); and organ system. FDA review staff who are responsible for traditional post-approval studies designs were consulted to determine counterfactual specifications. The counterfactual designs were devised using standards that have been established over years of FDA study design experience. The calculations for the hernia mesh counterfactual studies assumed prospective single-arm studies, with patients enrolling at 15 sites outside the United States, followed for 2 years with two in-person evaluations per year. For the robotic-assisted surgery device counterfactual study, we assumed a prospective cohort study with patients enrolling at 15 sites (50% of them from outside the US), followed for 30 days with one in-person evaluation [20].

### Return on investment, cost saving percentage, and time saved

The ROI and TS metrics were developed and used in previous publications applied to clinical specialty registries in other spaces [22, 23].

ROI—We defined the ROI as the cost of savings from investing in the ACHQC divided by the cost of investment in the ACHQC, multiplied by 100. The cost savings is the sum of costs of the observed analyses conducted in the ACHQC subtracted from the cost of the counterfactual studies.

CSP (Cost Savings Percentage)–This is equal to the difference between the cost of the counterfactual studies and the cost of the registry analyses (cost savings), divided by the cost of the counterfactuals, multiplied by 100.

**Fig. 1:**
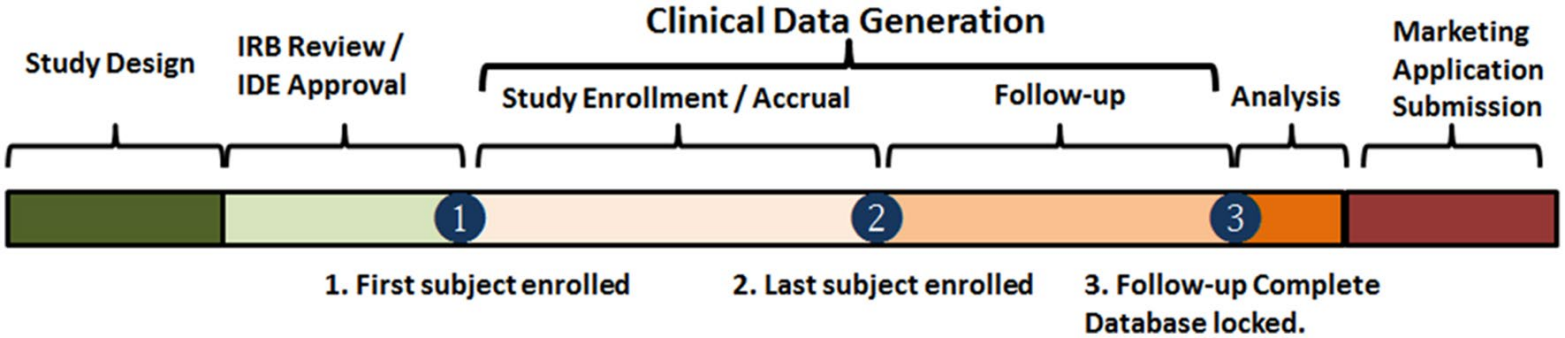
Time frame from study design to regulatory submission

TS—The time interval used for calculating the TS is the time duration from enrollment of the first patient in the study to the date that follow-up is complete on the last study participant, the clinical data generation period in Fig. 1.

The focus on the clinical data generation period frames a metric that can be meaningfully compared across and between registry-(RWE) studies and counterfactual studies, because it provides for standardization. The clinical data generation period does not include time for IRB approval given IRB approval can be variable; and time to enlist study sites, because in actual conduct of studies the time intervals may be combined and may overlap, making comparison between observed and counterfactual studies difficult.

TS using RWE was calculated by subtracting the time estimated for each counterfactual study from time needed to conduct the study using data found in the registry. The number of days needed for the observed analyses is the number of days specified in the study design (enrollment plus follow-up time). The time needed to do the analyses using ACHQC (enrollment plus follow-up time) was zero, because the data had already been collected at the time the analysis. For counterfactual studies, the estimate of time needed to complete a study is the sum of the time needed for enrollment and follow-up specified by the study design. The counterfactual study enrollment time (CET) was calculated by applying the rate of enrollment of the pivotal study (the premarket study for the product) to the specifications of the counterfactual study (study size and number of study sites called for by the design) using the following formula: CET = (number of patients for study by design / rate of enrollment) / number of study sites specified by design.

## Results

In total, 45,010 patients were analyzed including 41,112 patients for the first analysis (25 ACHQC reports compared to traditional post-approval studies (PAS)) and 3,898 patients for the second analyses (label expansion for robotic-assisted surgery) spanning data from 2013 to 2021. Results are presented separately for these two analyses.

### Analysis one: comparison of cost and time for ACHQC reports and traditional post-approval studies (PAS)

#### Cost comparison

Table 1 presents types of mesh comparing the costs of RWE (analyses using registry data) to estimated costs of traditional PAS for those products. Three companies paid the ACHQC a total of $814,873 to conduct the 25 analyses for 20 different mesh products (in the form of ACHQC reports and datasets) that were used to address the new EMA requirements for post-market surveillance of hernia mesh in the EU. This included data collected between 2013 and 2021. The real cost charged by ACHQC for analyses included two components: an average recurrent cost of the quality control reports plus an overhead that reflects an average cost of membership in ACHQC.

**Table 1:** Costs Comparisons for ACHQC Analyses and Counterfactual Studies for Mesh Products and a Robotic-Assisted Surgery Device: Abdominal Core Health Quality Collaborative (ACHQC)

Permanent synthetic mesh for 8 inguinal hernia analyses^a^	
Average real cost using RWE^ (*n* = 18,720)	$31,380
Cost per counterfactual study (*n* = 150)	$1,536,238
Permanent synthetic mesh for 14 ventral hernia analyses^a^	
Average real cost using RWE (*n* = 21,507)	$36,323
Cost per counterfactual study (*n* = 123)	$1,433,087
Resorbable synthetic mesh for 2 ventral hernia analyses^a^	
Average real cost using RWE (*n* = 735)	$12,870
Cost per counterfactual study (*n* = 294)	$2,086,376
Biologic mesh for 1 ventral hernia analysis^a^	
Real cost using RWE (*n* = 150)	$29,575
Cost of counterfactual study (*n* = 337)	$2,250,653
Total cost of 25 mesh analyses^b^	
Real cost using RWE (*n* = 41,112)	$814,873
Cost of counterfactual studies (*n* = 3,847)	$38,776,529
Robotic-assisted surgery device^c^	
Range of real cost of using RWE (*n* = 3,898)	$750,000–$2,000,000
Cost of counterfactual study (*n* = 600)	$4,207,784

^a^The calculations for the hernia mesh counterfactual studies are based on the model as described by Wimmer et al. (2016)

^b^The cost of real-world evidence (i.e., cost of the reports from ACHQC) are the sum of the cost of specific studies plus a factor to reflect cost of membership or overhead

^c^The cost of the study done in the registry for the robotic-assisted surgery device is presented as a range (the actual cost is proprietary information)

The estimated total cost for the 25 counterfactual studies was $38,776,529 (see Table 1). The difference between the cost of the 25 analyses using registry data and the counterfactual studies was approximately $38 million. The cost differences between RWE and traditional studies ranged between $1.3 and $2.2 million per study. The comparisons are offered heuristically–not as cost or time savings–because traditional post-approval studies were not required by EMA for these products. Nonetheless, had the ACHQC (or similar registry-based data) not been available, traditional post-approval studies would have been the chief means by which device manufacturers could produce adequate data for post-market surveillance.

#### Time comparison

Table 2 reflects the comparison of the time required to perform analyses in the ACHQC with the time that would have been needed to conduct a traditional PAS. The counterfactual time estimate ranged between 12 and 34 months for enrollment and was 24 months for subject follow-up. The differences between the real time and the counterfactual estimation times of conducting studies were between 3 and 4.8 years.

**Table 2:** Time comparisons for ACHQC registry analyses

Counterfactual studies^†^: size and time			Time difference between ACHQC analyses and counterfactual studies
Study size	Enrollment time	Observation time specified by design	
Permanent synthetic mesh for 8 inguinal hernia			
150	15 months	24 months	3.25 years
Permanent synthetic mesh for 14 ventral hernia			
123	12 months	24 months	3 years
Resorbable synthetic mesh for 2 ventral hernia			
294	29 months	24 months	4.45 years
Biologic mesh for 1 ventral hernia			
337	34 months	24 months	4.8 years
Robotic-assisted surgical device			
600	60 months	1 month	5.1 years

^a^And Counterfactual Studies^b^ For Mesh Products And Robotics Surgery Device: Abdominal Core Health Quality Collaborative (Achqc)

^a^The negligible time required for each ACHQC study are assigned zero, as the data was in the registry at the time of the ACHQC studies. The time difference calculated is then, the total of the enrollment time plus observation time of the counterfactual studies

^b^The counterfactual studies assume prospective enrollment of patients at 15 sites in the United States

### Analysis two: ROI for use of RWE for label expansion for robotic-assisted surgery

ACHQC data were used to conduct a retrospective cohort study of prospectively collected de-identified data to evaluate a robotic-assisted surgery device as part of a submission to the FDA for consideration of an expanded indication for ventral hernia repair [20]. The robotic analysis covered 4 years of data: 2013–2017. The individual patient follow-up was 30 days after hernia repair. Data from 3,898 patients were reviewed and 3566 patients propensity matched into three distinct analytic groups: (1) comparing robotic-assisted ventral hernia repair without myofascial release to open ventral hernia repair without myofascial release (1742 patients); comparing robotic-assisted ventral hernia repair without myofascial release to laparoscopic ventral hernia repair without myofascial release (1230 patients); and comparing robotic-assisted ventral hernia repair with myofascial release to open ventral hernia repair with myofascial release (594 patients). Data from 332 patients were also analyzed in an unmatched fashion comparing robotic-assisted repair to laparoscopic repair in a complex ventral hernia group. Several endpoints were assessed to evaluate the safety and effectiveness of robotic-assisted ventral hernia repairs including operating room time, return to operating room, transfusions, infections, conversion from robotic to open surgery, and early technical failures, supporting the FDA expansion of indications for use [20].

#### ROI

**Fig. 2:**
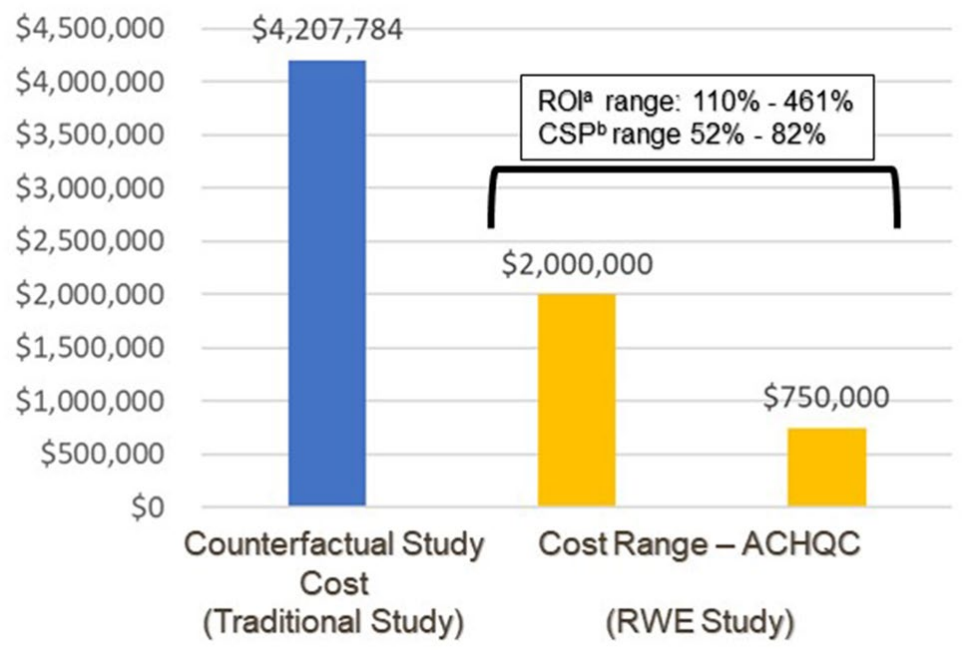
Return on Investment (ROI) and Cost Savings Percent (CSP) for use of Real-world Evidence (RWE) compared to Traditional Studies for Label Expansion of a Robotic-Assisted Surgery Device: Abdominal Core Health Quality Collaborative (ACHQC). The estimate of the cost of a counterfactual study is based on the Wimmer et al. model. The actual cost of the study used to support the label expansion is presented as a range. ^a^ROI is defined as the cost of savings from investing in the registry study (RWE) divided by the cost of investment in multiplied by 100. The cost savings is the sum of costs of the observed analyses conducted using RWE subtracted from the cost of the counterfactual studies. subtracted from the cost of observed analyses conducted in the ACHQC. ^b^Cost Savings Percentage (CSP) is equal to the difference between the cost of the counterfactual studies and the cost of the registry analyses (cost savings), divided by the cost of the counterfactuals, multiplied by 100.

Because the exact cost of the retrospective cohort study is confidential, we have used a range between $750,000 and $2 million for the cost in the ROI calculation; this range includes the actual cost of the evaluation (see Table 1).The estimated cost of the counterfactual premarket study would have been $4,207,784. The span of $2.2–$3.5 million savings represents a range of ROI 110–461%, or relative cost savings between 52 and 82%, respectively (Fig. 2).

#### TS

The time that would have been required to conduct the counterfactual study, the traditional study that might have been conducted if the registry did not exist, was calculated to be 5.1 years (Table 2). This estimation includes enrollment time to accrue sufficient cases plus the 30 days of follow-up.

## Discussion

This manuscript presents two analyses comparing RWE to evidence generated through traditional studies for regulatory use. In 25 analyses of 20 different mesh products, the cost and time required to produce traditional evidence was very high compared to use of data derived from the ACHQC. In a second analysis, a label expansion submission to the FDA for a robotic-assisted surgical device was supported by evidence also from the ACHQC. An ROI (between 110 and 461%) and substantial time savings (5 years) was documented based on the use of the ACHQC compared to a traditional PAS that would have been needed if the registry data had not been available. This 5-year Time Savings (TS) can best be appreciated by comparison to the 3–7 years needed to bring a device to market [24].

The model and assumptions used to calculate the ROI were likely underestimated for the following reasons. First, the cost estimate of the premarket counterfactual study (i.e., investigational study for labeling expansion) was made using these PAS assumptions produced by Wimmer et al. It is understood that premarket studies can be more expensive than PAS, due to premarket study needs thus suggesting the ROI is an underestimate. Second, the Wimmer et al. model was based on costs from prior to 2016. Adjusting for inflation to 2022 would increase these costs, and further underestimate the counterfactual study costs.

Regarding the robotic-assisted surgical device, besides savings in time and money, using the ACHQC data provided stronger evidence compared to traditional studies with public health benefit. The public health benefits of using the ACHQC stem from the larger study sizes frequently available using RWE. Nearly 4,000 cases from the registry were analyzed while we estimate 600 would have been needed for a traditional PAS. The larger numbers might allow study of heterogeneity of effect, study of outcomes for specific subpopulations, and for differences between operators and settings including groups for comparison of different treatments.

The study findings presented should be considered in the context of several study limitations. The analyses offered demonstrate the level of maturity of the ACHQC. A CRN maturity model evaluates the status of various registries and provides direction for future improvements [25]. The ACHQC is moderately mature compared to registries that have been evaluated using similar cost and time saving metrics. A key limitation of the time saving metric calculation is that the time for enrollment and for patient follow up in the ACHQC was zero. Certainly, time and effort were spent for data accrual and follow up, even though the data may have existed at the time of study creation. This would bias the time savings calculation in favor of ACHQC data use compared to prospectively collecting data. Specifically, ACHQC has low coverage (2% of all hernia operations in the U.S annually) and lack of systematic longitudinal follow-up beyond 30 days on all patients. This can lead to selection bias. Expanding registry coverage would provide increased cases for most products and support more robust analyses. As acceptance of participation in registries increases and the burden of data entry decreases, it is hoped that the ACHQC becomes more representative of U.S. hernia surgery in general. Currently, efforts are under way to integrate electronic medical record systems into the ACHQC and link ACHQC with Medicare claims data, and all-payer databases that will increase participation and longitudinal follow-up.

This demonstration of the value created by the ACHQC for evaluation and surveillance of hernia-related medical devices suggests that investment in the registry might improve evidence generation about current and future devices and procedures. Future directions based on this work include harmonization of registry data collection and outcomes on an international scale.

## Data Availability

The data are not available for analysis.
